# A randomized, double‐blind, placebo‐controlled, parallel‐group 12‐week pilot phase II trial of SaiLuoTong (SLT) for cognitive function in older adults with mild cognitive impairment

**DOI:** 10.1002/trc2.12420

**Published:** 2023-10-11

**Authors:** Genevieve Z. Steiner‐Lim, Alan Bensoussan, Elana R. Andrews‐Marney, Mahmoud A. Al‐Dabbas, Adele E. Cave, Christine L. Chiu, Katerina Christofides, Frances M. De Blasio, Lauren S. Dewsbury, Naomi L. Fagan, Jack S. Fogarty, Lena C. Hattom, Mark I. Hohenberg, Deyyan Jafar, Diana Karamacoska, Chai K. Lim, Jianxun Liu, Najwa‐Joelle Metri, D. Vincent Oxenham, Holly Ratajec, Nikita Roy, Danielle G. Shipton, David Varjabedian, Dennis H. Chang

**Affiliations:** ^1^ NICM Health Research Institute Western Sydney University Penrith New South Wales Australia; ^2^ Translational Health Research Institute (THRI) Western Sydney University Penrith New South Wales Australia; ^3^ Macquarie Medical School Macquarie University Macquarie Park New South Wales Australia; ^4^ Science of Learning in Education Centre National Institute of Education Nanyang Technological University Singapore; ^5^ School of Medicine Western Sydney University Penrith New South Wales Australia; ^6^ Xiyuan Hospital China Academy of Chinese Medical Sciences Beijing PR China; ^7^ Neuropsychology Department Royal North Shore Hospital St. Leonards New South Wales Australia; ^8^ School of Psychological Sciences, Faculty of Medicine, Health and Human Sciences Macquarie University Macquarie Park New South Wales Australia

**Keywords:** Alzheimer's disease, clinical trial, cognitive function, mild cognitive impairment, SaiLuoTong

## Abstract

**INTRODUCTION:**

This study primarily aimed to evaluate the efficacy and safety of SaiLuoTong (SLT) on cognition in mild cognitive impairment (MCI).

**METHODS:**

Community‐dwelling people with MCI aged ≥60 years were randomly assigned to 180 mg/day SLT or placebo for 12 weeks.

**RESULTS:**

Thirty‐nine participants were randomized to each group (*N* = 78); 65 were included in the final analysis. After 12 weeks, the between‐groups difference in Logical Memory delayed recall scores was 1.40 (95% confidence interval [CI]: 0.22 to 2.58; *P* = 0.010); Delis–Kaplan Executive Function System Trail Making Test Condition 4 switching and contrast scaled scores were 1.42 (95% CI: –0.15 to 2.99; *P* = 0.038) and 1.56 (95% CI: –0.09 to 3.20; *P* = 0.032), respectively; Rey Auditory Verbal Learning Test delayed recall was 1.37 (95% CI: –0.10 to 2.84; *P* = 0.034); and Functional Activities Questionnaire was 1.21 (95% CI: –0.21 to 2.63; *P* = 0.047; *P* < 0.001 after controlling for baseline scores).

**DISCUSSION:**

SLT is well tolerated and may be useful in supporting aspects of memory retrieval and executive function in people with MCI.

**Highlights:**

- SaiLuoTong (SLT) improves delayed memory retrieval and executive function in people with mild cognitive impairment (MCI).
- SLT is well tolerated in people ≥ 60 years.
- The sample of community dwellers with MCI was well characterized and homogeneous.

## 1 BACKGROUND

Mild cognitive impairment (MCI) is a heterogeneous syndrome characterized by cognitive decline and relatively preserved instrumental activities of daily living.^1^, ^2^ Conceptualized as the early symptomatic and clinically relevant prodromal phase of dementia, MCI increases dementia risk >5‐fold,^3^ with incident dementia estimated at ≈ 15% within 1 to 2 years,^4^, ^5^ and 65% to 80% within 3 to 6 years.^6^, ^7^, ^8^ Currently, there are no approved pharmacological interventions for MCI, and off‐label use of cholinesterase inhibitors is not recommended due to poor tolerability and safety.^5^, ^9^, ^10^, ^11^, ^12^

SaiLuoTong (SLT) is a modern compound Chinese herbal medicine preparation in capsule form containing standardized extracts of *Panax ginseng*, *Ginkgo biloba*, and *Crocus sativus* L at a 5:5:1 ratio. SLT has demonstrated various mechanisms of action relevant to MCI pathophysiology including anti‐inflammatory, antioxidant, antiapoptotic, antiplatelet aggregating, anti‐depressant, anxiolytic, as well as enhancing cholinergic function, reducing amyloid beta (Aβ), and increasing cerebral blood flow.^13^, ^14^, ^15^, ^16^, ^17^, ^18^, ^19^, ^20^, ^21^, ^22^, ^23^, ^24^ Studies in humans have demonstrated that SLT is safe, while improving cognitive function and activities of daily living in vascular dementia,^25^, ^26^ and modulating neurocognition in healthy young adults.^27^ It was hypothesized that a 12‐week treatment of SLT would improve cognitive function in older people with MCI compared to placebo.^28^

## 2 METHODS

Detailed methods have been published in the trial protocol^28^ and additional information can be found in supporting information.

### 2.1 Trial design and participants

This 12‐week randomized, double‐blind, placebo‐controlled, parallel group trial was conducted at NICM Health Research Institute, Western Sydney University, Australia. The study protocol was approved by Western Sydney University Human Research Ethics Committee (Approval H11878), registered with the Australian New Zealand Clinical Trials Registry (ACTRN12617000371392), and conducted in line with International Conference for Harmonisation Good Clinical Practice (ICH GCP) guidelines. Written, informed consent was obtained from each participant and their third‐party informant before commencing any screening activities.

Eligible participants who met all the study's inclusion criteria and none of the exclusion criteria were recruited from the community. Recruitment strategies in order of their success (from highest to lowest referrals) included a feature piece on primetime weekend morning television (Channel 9 Sydney *Today* show); a web and social media campaign through Trialfacts specialized patient recruitment services; university webpage listing of the study; referral from other concurrently running clinical trials; word of mouth; clinician referral; and flyers in public spaces (e.g., libraries), university campuses, and at residential aged care facilities.

Inclusion criteria included: aged ≥60 years, and meeting the core clinical criteria for MCI according to the National Institute on Aging–Alzheimer's Association (NIA‐AA) working group guidelines^1^ (see Section S1 in supporting information for how this was operationalized), including subjective memory complaints (corroborated by an informant), objective cognitive decline, no or minimal impairment in instrumental activities of daily living, and no severe depression. Exclusion criteria included: diagnosis of dementia or psychiatric disorders, history of drug and alcohol dependence or substance‐related disorders, seizures, or severe renal and hepatic disorders upon clinician review of pathology blood tests. Participants were reimbursed for travel expenses on a per‐visit basis ($30 each site visit) up to AUD$180. The full details of the inclusion and exclusion criteria are listed in Section S1 in supporting information.

### 2.2 Interventions

Full intervention details are in Table S1 in supporting information. Participants were randomly assigned to SLT or placebo groups for 12 weeks. Dose of SLT was 180 mg/day taken orally (4 × 45 mg capsules/day: 2 each morning and night). The placebo (4 capsules/day: 2 each morning and night) contained an inert substance matched for the color, taste, and smell of SLT (see Table S2 in supporting information). Capsules were manufactured by Shineway Pharmaceutical Group in a Good Manufacturing Practice facility certified by the Australian Therapeutic Goods Administration. Treatment compliance was monitored by counting returned capsules, participant completed medication diaries, and plasma concentrations of bioactive components of SLT and its metabolites (not reported here).

### 2.3 Outcomes

The primary outcome of interest that was used for sample size calculation was Logical Memory (Story A) delayed recall from the Weschler Memory Scale Fourth Edition (WMS‐IV).^29^ Three other primary outcomes included Coding from the Weschler Adult Intelligence Scale^®^ Fourth Edition (WAIS‐IV),^30^ Trail Making Test (TMT) Condition 4 from the Delis–Kaplan Executive Function System (D‐KEFS),^31^ and the Rey Complex Figure Test (RCFT).^32^ People with MCI who have deficits in episodic memory, executive function, and perceptual processing speed have a high risk of progressing to Alzheimer's disease (AD);^33^ accordingly,the primary outcomes were selected to test these cognitive domains. Logical Memory measures episodic memory recall, Coding measures perceptual processing speed, and D‐KEFS TMT Condition 4 and the RCFT measure aspects of executive function.

Secondary outcomes include the Montreal Cognitive Assessment (MoCA);^34^ the 21‐item Depression, Anxiety, Stress Scale (DASS‐21);^35^ the Quality of Life in Alzheimer's Disease (QoL‐AD) scale;^36^ the Functional Activities Questionnaire (FAQ);^37^ and the remaining neuropsychological test battery (see Table S3 in supporting information). These scales assess cognitive function, mood, quality of life, and instrumental activities of daily living, respectively. As shown in the schedule of activities (Table S3 in supporting information), these outcomes were assessed at baseline (week 0) and endpoint (week 12). Outcomes from the neuropsychological test battery were modified for online delivery (herein referred to as online protocol) for the final 10 trial participants due to COVID‐19 public health orders preventing face‐to‐face engagement with participants; modifications are detailed in Table S4 in supporting information. Blood samples were collected for apolipoprotein E (*APOE*) ε4 genotyping and the protocol is detailed in Section S2 in supporting information.

**Research in Context**

1. **Systematic review**: Databases (e.g., Web of Science) and clinical trial registries (e.g., ANZCTR) were systematically reviewed on August 16, 2022 for clinical trials assessing SaiLuoTong (SLT) for mild cognitive impairment (MCI). While there were published trials in other populations (e.g., vascular dementia), none were found for MCI. These relevant studies are appropriately cited.
2. **Interpretation**: This trial demonstrates that 12 weeks of SLT can improve delayed episodic memory retrieval and cognitive concept switching as a measure of executive function in people with MCI, while being safe and well tolerated. The psychometric outcomes should be mapped to in vivo trial effects on pathology relevant to MCI and SLT's mechanisms of action.
3. **Future directions**: Another trial is required with a larger sample size, longer treatment duration, and dose‐determination that also measures MCI pathology.

Safety was monitored via adverse event (AE) and serious adverse event (SAE) reporting and routine pathology blood tests for the duration of the study at pre‐defined intervals detailed in the study protocol. AEs were classified according to the National Cancer Institute (NCI) common terminology criteria for adverse events (CTCAE) version 5.^38^ This framework describes and categorizes AEs within system organ classes (SOCs). SOCs of concern include gastrointestinal disorders; general disorders and administration site conditions; musculoskeletal and connective tissue disorders; metabolism and nutrition disorders; nervous system disorders; immune system disorders; psychiatric disorders; skin and subcutaneous tissue disorders; infections and infestations; and respiratory, thoracic, and mediastinal disorders. AEs within each SOC were specified.

### 2.4 Sample size

Sample size was calculated a priori to be 63 across two parallel groups based on achieving an effect size of Cohen *f* = 0.36 at *α* = 0.05 and 80% power on the WMS‐IV Logical Memory Story A delayed recall following Craft.^39^ Accounting for a 20% dropout, 76 participants were required, which was then rounded to 80 (40 per group) to facilitate the randomization strategy (below).

### 2.5 Randomization

A permuted block randomization strategy (block size = 4) was used to generate the randomization sequence by a university staff member external to the research team using Microsoft Excel. The 1:1 allocation was concealed using 10 unique randomly generated batch numbers (5 per group) that were sent directly to the manufacturer. Enrolled participants were then allocated to the next sequentially numbered box of investigational product by a member of the research team. All participants, researchers, outcome assessors, and data analysts were blinded to the group allocation.

### 2.6 Statistical methods

All statistical analyses were conducted in Stata 17.0 (StataCorp). All variables were visually inspected for normality via histogram, and skew and kurtosis were investigated using the criterion |*z*| > 2.58 (*P* > 0.010). To compare participant baseline characteristics, two‐tailed independent groups *t* tests with equal variances assumed were conducted for all continuous variables (age, education, premorbid function, global cognition, depression, body mass index, and lost to follow‐up), and chi‐squared tests were conducted for categorical variables (sex, psychoactive medication use, and *APOE* ε4 carrier status).

Independent‐groups *t* tests with equal variances assumed were used to assess the group differences (SLT vs. placebo) in the mean change (endpoint minus baseline) of primary and secondary outcomes. Participant baseline characteristics detailed above were checked as confounders against all variables and controlled for, if appropriate, using analysis of covariance. Missing data were accounted for by conducting complete case analysis. Because the study hypothesis was directional, all tests were conducted one‐tailed, *α* = 0.05. Mean differences, standard deviations, 95% confidence intervals, and effect sizes are reported. Further, it should be noted that because this trial details the results for multiple dependent variables, the frequency of Type I errors increases. However, Howell^40^ argues that this increase in *frequency* of Type I errors cannot be controlled by adjusting *α* levels, because the *probability* of Type I error remains the same (i.e., at 5% according to our *α* level).

## 3 RESULTS

### 3.1 Study participants

**FIGURE 1:**
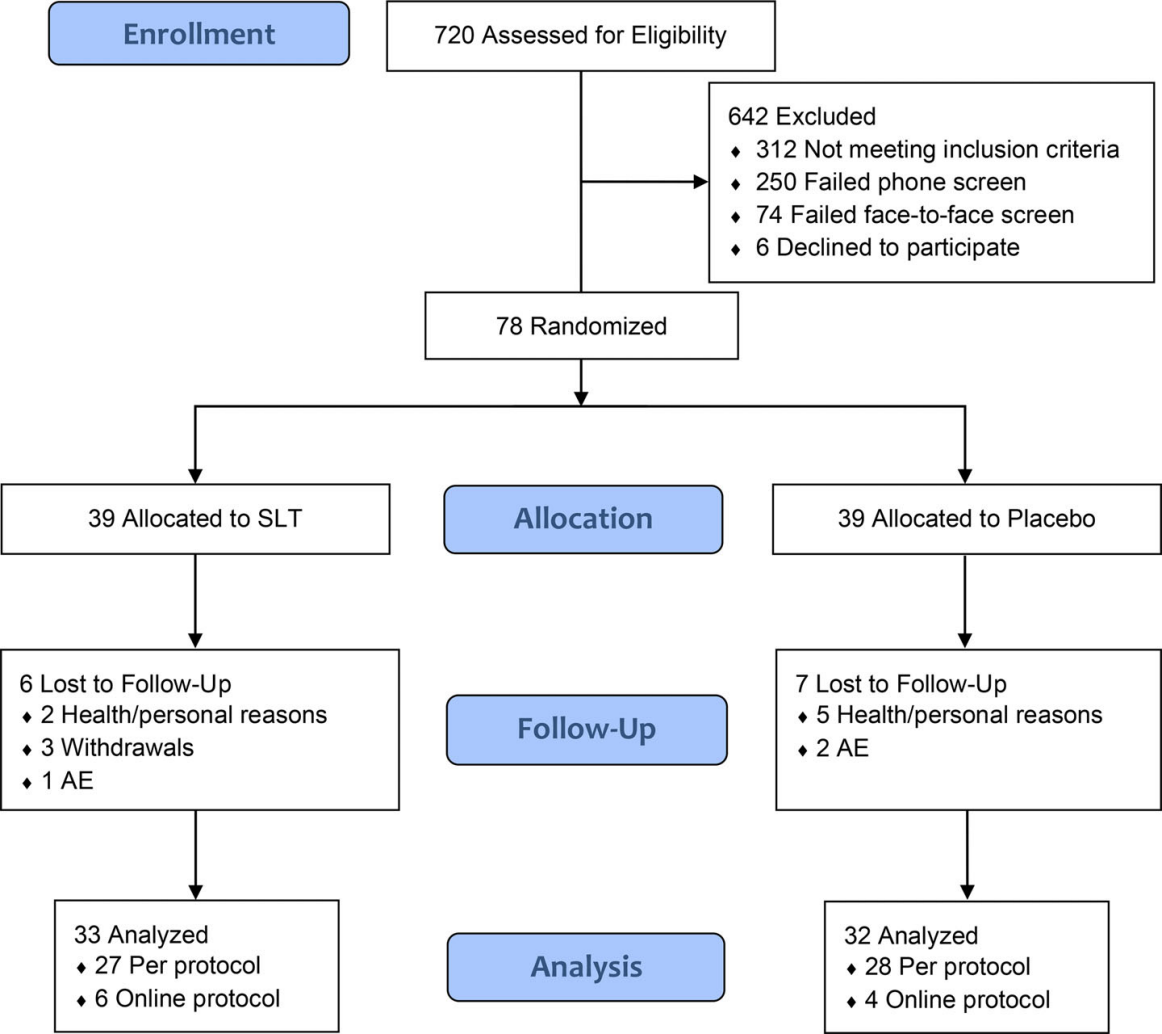
Schematic of participant flow including number of participants at each stage of the trial. AE, adverse event; SLT, SaiLuoTong.

A diagram of participant flow is detailed in Figure 1. During the study enrolment period (April 3, 2017 to February 27, 2020), 720 potential participants contacted the research team, of which 408 were screened via telephone, 158 were screened face to face, before 78 were enrolled and randomized to receive either SLT (*n* = 39) or placebo (*n* = 39). There were 13 participants lost to follow‐up (SLT *n* = 6; placebo *n* = 7) with reasons specified in Figure 1. A total of 65 completed the study (SLT *n* = 33; placebo *n* = 32) and were analyzed for the primary outcome, representing a 16.7% dropout rate. Of these, 10 completed the online protocol for their endpoint assessment (at week 12) due to restrictions limiting face‐to‐face contact because of COVID‐19 public health orders. Last patient, last visit (*N* = 78) was achieved May 26, 2020, after which the trial was closed because of COVID‐19 restrictions.

Participant demographics and clinical characteristics at baseline are detailed in Table 1. Instrumental activities of daily living (ADLs), as measured by the FAQ, were slightly better in the SLT compared to the placebo group at baseline (*M _diff_* = −1.2, 95% confidence interval [CI: −2.2, −0.2]); this was well below the study's cut‐off range for inclusion at 9 points. The groups did not differ significantly on any other measure at baseline.

**TABLE 1:** Demographic and clinical characteristics for SLT and placebo groups at baseline.

Participant characteristics	SLT (*n* = 39)	Placebo (*n* = 39)	*P*‐value
Age, years mean ± SD	69.1 ± 6.0	71.5 ± 6.9	0.100
Sex, F:M (% female)	23:16 (59%)	18:21 (46%)	0.257
*APOE* ε4 status^a^, pos:neg (% pos)	19:19 (50%)	17:21 (45%)	0.646
Education, years mean ± SD	14.8 ± 3.7	14.4 ± 3.4	0.587
Premorbid function, ToPF mean ± SD	106.8 ± 11.4	105.8 ± 12.7	0.723
Global cognition, MoCA mean ± SD	24.3 ± 3.4	23.4 ± 3.0	0.228
Instrumental ADLs, FAQ	1.8 ± 1.4	3.1 ± 2.7	**0.016** ^*^
Depression, GDS mean ± SD	7.7 ± 5.2	8.6 ± 4.5	0.444
BMI, mean ± SD	29.6 ± 6.4	28.3 ± 5.8	0.345
Alcohol, mean units/week ± SD	6.2 ± 7.7	7.3 ± 7.7	0.548
Psychoactive medications, Y:N (% yes)	11:27 (29%)	17:22 (44%)	0.182
Lost to follow‐up, *N* (%)	6 (15%)	7 (18%)	0.761
Compliance, %	96.7 ± 7.2	97.0 ± 5.4	0.711

*Note*: Psychoactive medications include anxiolytics and anti‐depressants. Compliance calculated as the differences between the expected number (e) of capsules returned and the actual capsules (a) returned relative to the total number of capsules dispensed (d): e‐a/d.

Abbreviations: ADLs, activities of daily living; *APOE*, apolipoprotein E; BMI, body mass index; FAQ, Functional Activities Questionnaire; GDS, Geriatric Depression Scale; MoCA, Montreal Cognitive Assessment; SD, standard deviation; SLT, SaiLuoTong; ToPF, Test of Premorbid Function actual score.

^a^ *N* = 38 per group as blood could not be collected from two participants for genotyping. *APOE* ε4 positivity was to be determined if participants carried one or more copies of the ε4 allele, with all participants in the final sample carrying a maximum of one copy.

* Bolded *P* < 0.05, two‐tailed.

### 3.2 Analyses of primary and secondary outcome measures

Upon inspection, all data were found to be normally distributed, thus parametric analyses ensued. There was no evidence of confounding from baseline participant characteristics, other than for the FAQ (see Table 1). It was thus unnecessary to control for baseline participant characteristics in subsequent analyses of all other outcome measures.

**FIGURE 2:**
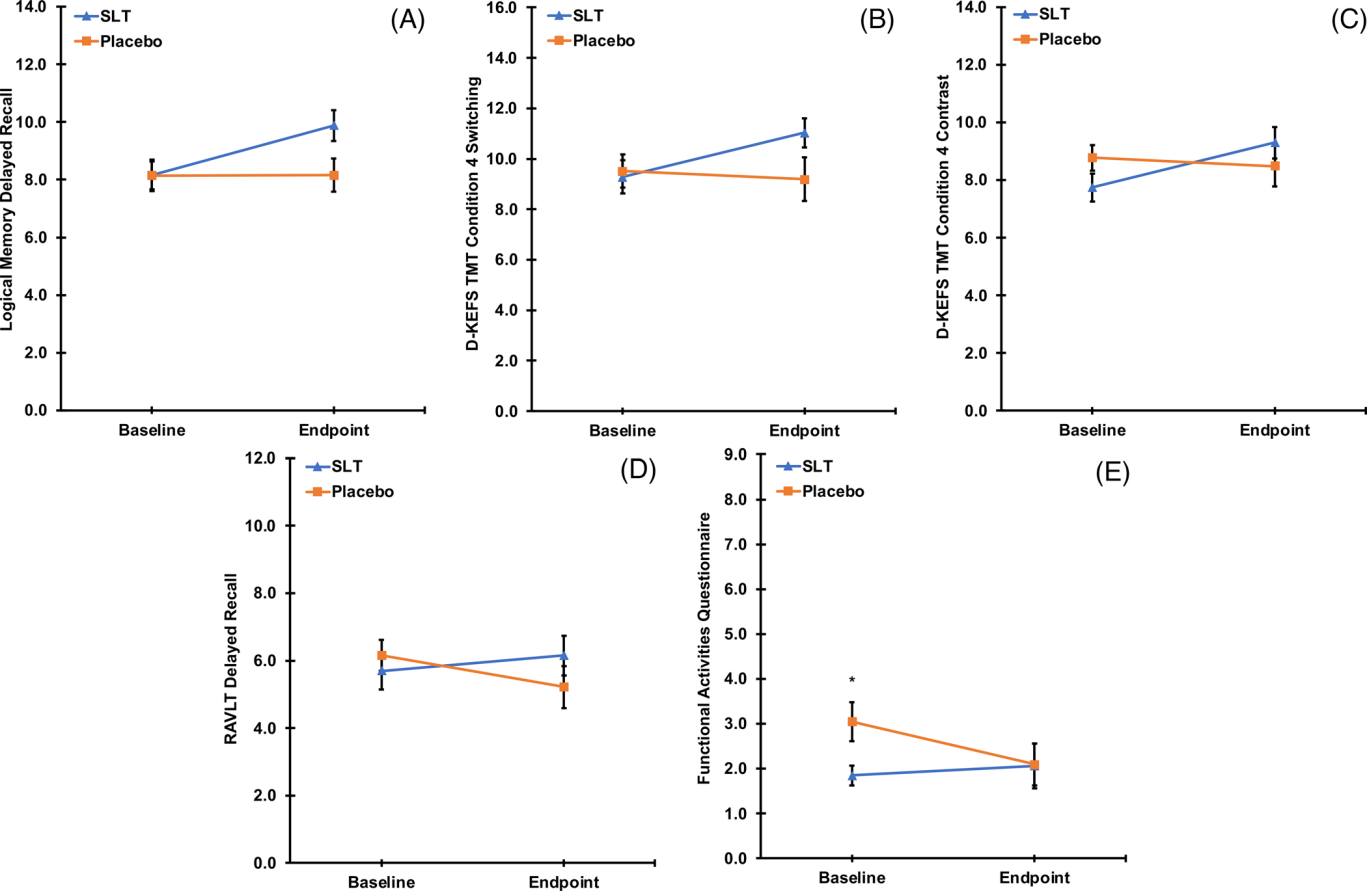
Statistically significant outcomes from the mean difference data shown in Table 2 are visualized here using line charts showing the separate means and standard errors for each group (SaiLuoTong [SLT] = blue; placebo = orange) and timepoint (baseline and endpoint). Outcome measures include: Wechsler Memory Scale Fourth Edition Logical Memory delayed recall score (A), Delis–Kaplan Executive Function System Trail Making Test condition 4 switching (B) and contrast (C) scaled scores, Rey Auditory Verbal Learning Test delayed recall score (D), and Functional Activities Questionnaire (FAQ; E) scores. * Denotes *P* < 0.05 for FAQ scores at baseline.

Table 2 details the outcomes and estimations for all primary and secondary outcomes, including mean change (endpoint minus baseline), standard deviation, and 95% CI for SLT and placebo groups. Mean difference between groups (SLT minus placebo) and the 95% CI, *P*‐value (starred* for statistical significance at *P* < 0.05, one‐tailed) and degrees of freedom from the independent groups *t* tests, and Cohen's *d* are also shown. Positive difference values indicate an improvement in all variables other than for the FAQ and DASS subscales, in which negative differences indicate an improvement (higher positive scores = poorer outcomes). Missing data were as follows: Block Design (*N_missing_* = 10), D‐KEFS TMT (*N_missing_* = 4), Coding and Benton Visual Retention Test (each *N_missing_* = 1); complete cases (*N* = 65) were analyzed for all other outcome measures. Figure 2 displays the means and standard errors for each group and timepoint for statistically significant outcome measures only.

**TABLE 2:** Mean change (endpoint minus baseline), between‐group differences (SLT minus placebo), statistical comparison, and effect size for each of the primary and secondary outcome measures for SLT and placebo groups.

	Mean change (endpoint – baseline)		Between‐group difference		
	SLT mean difference ± SD [95% CI]	Placebo mean difference ± SD [95% CI]	SLT – Placebo mean difference [95% CI]	*P*‐value	Cohen's *d*
**Primary outcome measures**					
Logical Memory delayed recall (20 min)^a^	1.24 ± 2.21 [0.46, 2.03]	–0.16 ± 2.54 [−1.07, 0.76]	1.40 [0.22, 2.58]	**0.010** ^*^	0.59
D‐KEFS TMT condition 4					
Switching	1.00 ± 3.09 [−0.15, 2.15]	–0.42 ± 3.04 [−1.54, 0.70]	1.42 [−0.15, 2.99]	**0.038** ^*^	0.46
Contrast	1.17 ± 3.45 [−0.12, 2.46]	–0.39 ± 2.96 [−1.47, 0.70]	1.56 [−0.09, 3.20]	**0.032** ^*^	0.48
Coding	0.41 ± 1.41 [−0.10, 0.91]	0.47 ± 1.52 [−0.08, 1.02]	–0.06 [−0.80, 0.67]	0.567	0.04
RCFT					
Copy	–0.94 ± 5.33 [−2.83, 0.95]	–1.41 ± 5.98 [−3.56, 0.75]	0.47 [−2.34, 3.27]	0.370	0.08
Delayed Recall	0.95 ± 5.28 [−0.92, 2.83]	1.19 ± 5.92 [−0.95, 3.32]	–0.23 [−3.01, 2.55]	0.566	0.04
**Secondary outcome measures**					
MoCA	0.15 ± 3.45 [−1.07, 1.37]	0.00 ± 3.59 [−1.30, 1.30]	0.15 [−1.59, 1.90]	0.431	0.04
FAQ	0.24 ± 2.85 [−0.77, 1.25]	–0.97 ± 2.89 [−2.01, 0.07]	1.21 [−0.21, 2.63]	**0.047** ^*^	0.42
QoL‐AD	0.91 ± 4.03 [−0.52, 2.34]	–0.66 ± 4.76 [−2.37, 1.06]	1.57 [−0.62, 3.75]	0.079	0.36
DASS‐21					
Depression scale	–0.30 ± 2.56 [−1.21, 0.60]	–0.22 ± 2.60 [−1.16, 0.72]	–0.08 [−1.36, 1.19]	0.552	0.03
Anxiety scale	0.06 ± 1.95 [−0.63, 0.75]	0.06 ± 2.33 [−0.78, 0.90]	0.00 [−1.07, 1.06]	0.501	0.00
Stress scale	–0.33 ± 2.20 [−1.11, 0.45]	–0.75 ± 3.35 [−1.96, 0.46]	0.42 [−0.98, 1.82]	0.277	0.15
RAVLT					
Trial I–V Total	–1.58 ± 9.01 [−4.77, 1.62]	–2.69 ± 8.81 [−5.86, 0.49]	1.11 [−3.31, 5.53]	0.308	0.12
Trial B	–0.18 ± 1.53 [−0.72, 0.36]	–0.19 ± 1.73 [−0.81, 0.44]	0.01 [−0.80, 0.81]	0.494	0.00
Trial VI	0.39 ± 2.62 [−0.54, 1.32]	–0.50 ± 2.18 [−1.29, 0.29]	0.89 [−0.30, 2.09]	0.071	0.37
Delayed recall (20 min)	0.21 ± 3.38 [−0.99, 1.41]	–1.16 ± 2.46 [−2.04, −0.27]	1.37 [−0.10, 2.84]	**0.034** ^*^	0.46
Logical Memory immediate recall	1.12 ± 2.07 [0.39, 1.86]	0.75 ± 2.64 [−0.20, 1.70]	0.37 [−0.80, 1.55]	0.265	0.16
D‐KEFS TMT condition 2	0.33 ± 2.56 [−0.62, 1.29]	0.65 ± 3.38 [−0.60, 1.89]	–0.31 [−1.85, 1.23]	0.657	0.10
Digit Span					
Forward	0.06 ± 2.77 [−0.92, 1.04]	0.88 ± 2.98 [−0.20, 1.95]	–0.81 [−2.24, 0.61]	0.871	0.28
Backward	–0.12 ± 3.30 [−1.29, 1.05]	0.44 ± 2.76 [−0.56, 1.43]	–0.56 [−2.07, 0.95]	0.769	0.18
Total	0.16 ± 1.97 [−0.55, 0.85]	0.41 ± 2.56 [−0.52, 1.33]	–0.25 [−1.39, 0.88]	0.673	0.11
Block Design	0.85 ± 2.43 [−0.11, 1.81]	–0.11 ± 2.51 [−1.08, 0.87]	0.96 [−0.38, 2.30]	0.078	0.39
Benton Visual Retention Test	–0.53 ± 2.53 [−1.44, 0.38]	–0.53 ± 2.17 [−1.31, 0.25]	0.00 [−1.18, 1.18]	0.500	0.00
Boston Naming Test	–0.79 ± 1.05 [−1.16, −0.41]	–0.34 ± 1.36 [−0.83, 0.15]	–0.44 [−1.05, 0.16]	0.928	0.37
Semantic fluency	–0.52 ± 3.47 [−1.74, 0.71]	–1.28 ± 6.06 [−3.47, 0.90]	0.77 [−1.67, 3.20]	0.266	0.16
COWAT	–0.97 ± 3.09 [−2.06, 0.12]	–0.88 ± 3.84 [−2.26, 0.51]	–0.09 [−1.82, 1.63]	0.544	0.03

*Note*: Scaled scores are reported where available (D‐KEFS TMT, Block Design, Digit Span, Coding). The Osterrieth scoring system was used for the RCFT.

Abbreviations: CI, confidence interval; COWAT, Controlled Oral Word Association Test; D‐KEFS, Delis–Kaplan Executive Function System; DASS‐21, 21‐item Depression, Anxiety, Stress Scale; FAQ, Functional Activities Questionnaire; MoCA, Montreal Cognitive Assessment; RAVLT, Rey Auditory Verbal Learning Test; RCFT, Rey Complex Figure Test; QoL‐AD, Quality of Life in Alzheimer's Disease scale; SD, standard deviation; SLT, SaiLuoTong; TMT, trail making test.

^a^ Primary outcome measure on which sample size calculation was based.

* **Denotes** *P* < 0.05, one‐tailed.

The mean change in the primary outcome measure used for sample size estimation, Logical Memory delayed recall, was found to significantly increase after administration of SLT compared to placebo, with a moderate effect size. Change in both the switching and contrast scaled scores for D‐KEFS TMT Condition 4 were also found to significantly increase with SLT versus placebo; both with moderate effect sizes. There was no significant statistical difference associated with SLT treatment in WAIS‐IV Coding or RCFT scores for both copy and delayed recall conditions.

As shown in Table 2 and Figure 2E, the mean change in FAQ score differed significantly between groups and was driven by a reduction (improvement) in the placebo compared to SLT group, with a moderate effect size. This effect remained after controlling for baseline FAQ scores, *F*(3, 61) = 6.98, *P* < 0.001. Change in RAVLT delayed recall scores also differed between groups, which was driven by a reduction in the placebo group (as can be seen in Figure 2D); a moderate effect size was observed. As detailed in Table 2, all other secondary outcomes were not statistically significant.

### 3.3 Safety

In total, 22 participants (28%) experienced at least one AE during the trial intervention period (SLT *n* = 12, 15%; placebo *n* = 10, 13%). Overall, 39 AEs were reported (SLT *n* = 21, 54%; placebo *n* = 18, 46%), 17 of which were classified as mild (44%), 20 as moderate (51%) and 2 as severe (5%). A total of 9 AEs (23%) had possible causality attributed to the study treatment; however, upon unblinding, only three of these (8%) were found to be in the SLT group across two participants. There were three withdrawals after an AE, of which two were from the SLT group, and only one of these had causality attributed to the study treatment; this was due to abdominal pain and back pain.

Table 3 details the breakdown of these AEs and SAEs according to their expectancy (expected vs. unexpected) reported between baseline and midpoint (weeks 0–6) and between midpoint and endpoint (weeks 6–12). Gastrointestinal upset, including nausea, abdominal pain, and reflux, was the most common AE; the number of these events was comparable between groups (SLT *n* = 8; placebo *n* = 7). The two SAEs recorded were found to have occurred in the placebo group.

**TABLE 3:** The frequency of expected and unexpected adverse events (AEs) and serious adverse events (SAEs) for SLT and placebo groups reported between baseline–midpoint (week 0–6) and midpoint–endpoint (week 6–12).

	SLT (*n* = 33)		Placebo (*n* = 32)	
	Week 0–6	Week 6–12	Week 0–6	Week 6–12
**Total *N* AEs/SAEs (%)**	13 (33%)	8 (21%)	13 (33%)	5 (13%)
**Expected AEs**				
Headache	2			1
Fatigue	1			
Dizziness	1	1		
GI upset^*^	5	3	6	
**Unexpected AEs**				
Musculoskeletal^**^	1	2	1	1
Increased blood glucose			1	
Infections and infestations				1
Rash maculo‐papular			1	
Allergic reaction	1	1		1
Respiratory			1	
Neurological	1			1
Mental health^***^	1	1	1	
**SAEs**				
GI upset*	0	0	2	0
**Drug related AEs resulting in treatment discontinuation**				
‐	0	1	2	0

Abbreviations: GI, gastrointestinal; SLT, SaiLuoTong.

* GI Upset refers to AEs abdominal pain, constipation, diarrhea, gastroesophageal reflux, and nausea.

** Musculoskeletal refers to AEs muscle pain and bone pain.

*** Mental Health refers to AEs including depression and anxiety.

## 4 DISCUSSION

This was the first study to assess the effects of SLT on cognition in people with MCI.^25^, ^26^, ^27^ Significant improvements after 12 weeks of SLT compared to placebo were seen in the primary outcome measure scores for Logical Memory delayed recall and D‐KEFs TMT Condition 4. Together, this indicates enhanced delayed episodic memory retrieval and executive function pertaining to switching between cognitive concepts, higher‐level divided attention, and multitasking (both before and after accounting for the influence of processing speed) with SLT treatment. After 12 weeks in the placebo group, RAVLT delayed recall scores were lower compared to SLT, suggesting a maintenance of the spontaneous delayed retrieval of unstructured verbal information with SLT.

FAQ scores improved in the placebo group after controlling for baseline differences. This is an unexpected finding and differs from previous studies involving people with vascular dementia that reported improvements in ADLs with SLT^25^, ^26^ after longer dosing regimens (16–26 weeks). Nevertheless, it should be noted that the 1.21 point difference in FAQ change scores is not considered clinically relevant as it falls below the 3‐ to 5‐point difference that is considered clinically meaningful.^31^ No other differences in primary or secondary outcomes after 12 weeks SLT (vs. placebo) were observed.

Collectively, results suggest that SLT may be useful in supporting memory retrieval and executive function in people with MCI. SLT appears to have select sensitivity to delayed recall measures of verbal memory retrieval in MCI, with improvements noted in Logical Memory and RAVLT delayed recall scores, but not in RCFT delayed recall scores (which reflects non‐verbal memory retrieval and visuospatial construction). However, the functional significance and clinical relevance of these effects is currently unknown given that the mean differences observed were relatively modest (1.37–1.56), with moderate effect sizes (Cohen *d* = 0.46–0.59). Further investigation to understand whether these findings have an impact on maintaining or improving day‐to‐day function, and thus quality of life, is required.

Our finding that SLT improved memory retrieval and executive function was largely consistent with improvements in global cognition observed in people with vascular dementia after 16 weeks^26^ and 52 weeks of treatment with SLT,^25^ and neurocognitive changes observed in healthy adults after 1 week of SLT.^27^ Both individually and in combination, the bioactive compounds in SLT derived from *Panax ginseng*, *Ginkgo biloba*, and *Crocus sativa* L have been shown to have therapeutic potential against pathogenic mechanisms associated with MCI, AD, and vascular dementia.^13^, ^14^, ^15^, ^16^, ^17^, ^18^, ^19^, ^20^, ^21^, ^22^, ^23^, ^24^ SLT's antioxidant, anti‐inflammatory, antiapoptotic, and antiplatelet aggregating properties, as well as its capacity to increase cerebral blood flow and cholinergic function, and reduce Aβ accumulation, may underpin the cognitive effects of SLT in MCI observed here;^13^, ^15^, ^16^, ^19^, ^23^, ^24^ the mechanism is currently being explored in several substudies as detailed in our trial protocol.^28^

Gastrointestinal upset was reported at a similar frequency in both the SLT and placebo group (21% vs. 15%, respectively) and most AEs were classified as either mild or moderate (95%). Compliance was high across groups (>96%) and there were only three withdrawals after an AE in the SLT group. Together, this supports the existing evidence that SLT is generally well tolerated and can be considered safe for the treatment of cognitive problems in older people.^25^, ^26^, ^41^

A major strength of this trial was its well characterized and homogeneous sample due to how we operationalized the NIA‐AA core clinical criteria with our eligibility criteria. We are confident that this sample is representative of people with MCI who have an increased risk of AD as the prevalence of *APOE* ε4 in 47% of this sample is consistent with the Australian Imaging, Biomarker, & Lifestyle Flagship Study of Ageing (AIBL), which reported 46% *APOE* ε4 positivity in people with MCI (carrying ε4 allele; *N* = 150).^42^ Compared to the estimated global prevalence of ≈ 14% in cognitively normal individuals,^42^, ^43^ the similarity in *APOE* ε4 positivity in the current study and the AIBL cohort indicates the generalizability of our findings to other MCI cohorts. Future work may also seek to apply the amyloid/tau/neurodegeneration criteria^44^ with an additional criterion for cerebrovascular load,^25^, ^45^ or assess a vascular MCI population, particularly given the known mode of action for SLT targeting vascular pathology. Other strengths included succeeding the minimum sample size of *N* = 63, providing enough power to detect an effect, and the successful randomization protocol given minimal baseline differences between SLT and placebo groups.

This trial was not without limitations including the potential source of measurement bias that could have been introduced when completing the online protocol for the final 10 participants (SLT *n* = 6; placebo *n* = 4) enrolled in the trial due to COVID‐19 (see Table S4). The choice of primary outcome for sample size calculation was appropriate for the cohort. However, the findings are difficult to interpret given that there is no established minimal important clinical difference for the Logical Memory delayed recall. Nonetheless, our participants fell within the MCI range used by other cohorts (e.g., Alzheimer's Disease Neuroimaging Initiative 2) for participants with a similar level of education (5–11 points).^46^ Future trials could seek to adopt widely used measures such as the Alzheimer's Disease Assessment Scale‐Cognitive subscale or Clinical Dementia Rating Sum of Boxes as secondary endpoints for added comparability between studies and aid with determining clinical significance, or even create a delayed verbal recall composite endpoint. Further, MCI was characterized clinically, but future studies could seek to use an appropriate pathology marker as entry criteria (e.g., Aβ positivity) and measure changes in response to SLT treatment.

In conclusion, our findings suggest that for people with MCI, 12 weeks of treatment with SLT compared to placebo can improve delayed episodic memory retrieval and executive function including cognitive concept switching, higher‐level divided attention, and multitasking in people with MCI. Future research should seek to elucidate the clinical relevance of these effects and map these psychometric outcomes to in vivo trial effects on pathology relevant to MCI and SLT's mechanisms of action. Another trial with a larger sample size, longer treatment duration, and dose determination is required.

## AUTHOR CONTRIBUTIONS

Genevieve Z. Steiner‐Lim was the lead investigator and responsible for all aspects of the trial including protocol design and drafting, overseeing the execution of the protocol and trial conduct, data collection, data reporting and analysis, and publication of results. Dennis H. Chang and Alan Bensoussan contributed to the conception and design of the study. Elana R. Andrews‐Marney, Naomi L. Fagan, David Varjabedian, and Diana Karamacoska contributed to the study execution and management of the protocol. Elana R. Andrews‐Marney, Mahmoud A. Al‐Dabbas, Adele E. Cave, Naomi L. Fagan, Jack S. Fogarty, Lena C. Hattom, Deyyan Jafar, Nikita Roy, Danielle G. Shipton, and David Varjabedian contributed to data acquisition. Christine L. Chiu, Katerina Christofides, Frances M. De Blasio, Lauren S. Dewsbury, Jack S. Fogarty, Deyyan Jafar, Chai K. Lim, Najwa‐Joelle Metri, Holly Ratajec, and David Varjabedian assisted with data reporting and analyses. Jianxun Liu developed and standardized the intervention being used. Mark I. Hohenberg, Deyyan Jafar, and D. Vincent Oxenham advised on medical information, recruitment, and clinical issues. Mahmoud A. Al‐Dabbas and David Varjabedian contributed to trial auditing and International Conference of Harmonization Good Clinical Practice compliance. Genevieve Z. Steiner‐Lim drafted the final manuscript, and all authors provided feedback, read, and approved the final version of the manuscript to be published and agree to take responsibility for all aspects of the research to ensure accuracy and integrity.

## CONFLICT OF INTEREST STATEMENT

As a medical research institute, NICM Health Research Institute receives research grants and donations from foundations, universities, government agencies, individuals, and industry. Sponsors and donors provide untied funding for work to advance the vision and mission of the Institute. The project that is the subject of this article was not undertaken as part of a contractual relationship with any organisation other than the funding declared. Author disclosures are available in the supporting information.

## CONSENT STATEMENT

All participants and their third‐party informant provided written informed consent before commencing any screening activities.

## Supporting information

Supplementary Materials

Supplementary Materials
