# Supplementary material for: A randomized, double‐blind, placebo‐controlled, parallel‐group 12‐week pilot phase II trial of SaiLuoTong (SLT) for cognitive function in older adults with mild cognitive impairment
Source: Alzheimers Dement (N Y). 2023 Oct 11;9(4):e12420. doi: 10.1002/trc2.12420 (PMC10565903; doi:10.1002/trc2.12420)
Supplement: Supplementary file 1 — Supplementary Materials [file TRC2-9-e12420-s001.docx]

**Supplementary Materials**

| **Table s1.** Quantities for the dry powder extract constituents of the SLT formulation contributing to the 180 mg daily dose. | |
| --- | --- |
| **Standardised Herbal Extracts** | **Daily Quantity** |
| *Panax ginseng* standard extract  Ginsenosides Re, Rg1, Rb1 | 81.8 mg |
| *Ginkgo biloba* standard extract  Rutin, flavonol glycosides (quercetin, kaempferide, isorhamnein), terpene lactones (ginkgolide A, B, C, bilobalide) | 81.8 mg |
| *Crocus sativa* L (stigma croci) standard extract  Croci glycosides-I | 16.4 mg |
| ***Total*** | 180.0 mg |

| **Table s2.** Constituents and their proportions for the placebo formulation. | |
| --- | --- |
| **Ingredient** | **Proportion/Quantity** |
| *Major constituents* |  |
| Mixed starch | 44.8 % |
| Dried carrot powder | 55.2 % |
| *Minor constituents contributing to <1.06 %* |  |
| Sunset yellow 85020 | 30 ppm |
| Tartrazine 60 | 20 ppm |
| Powder flavour | 0.55 % |
| Denatonium benzoate | 0.0008 % |
| Burnt sugar colour | 0.50 % |
| *Note:* ppm = parts per million | |

| **Table s3.** Schedule of activities. | | | | | | |
| --- | --- | --- | --- | --- | --- | --- |
| **Testing schedule** | **Task** | **Phone**  **Screen** | **Face-to-Face**  **Screen** | **Baseline**  **(0 weeks)** | **Midpoint**  **(6 weeks)** | **Endpoint**  **(12 weeks)** |
| Screening | Eligibility Criteria | X |  |  |  |  |
|  | TICS-M | X |  |  |  |  |
|  | Medical History |  | X |  |  |  |
|  | IQCODE |  | X |  |  |  |
|  | TOPF (part phone/F2F) | X | X |  |  |  |
|  | MoCA* |  | X |  |  | X |
|  | FAQ* |  | X |  |  | X |
|  | GDS |  | X |  |  |  |
|  | RAVLT* |  | X |  |  | X |
| Primary outcomes | WMS-IV Logical Memory |  |  | X |  | X |
|  | D-KEFS TMT Condition 4 |  |  | X |  | X |
|  | WAIS-IV Coding |  |  | X |  | X |
|  | Rey Complex Figure Test |  |  | X |  | X |
| Secondary outcomes | QOL-AD |  |  | X |  | X |
|  | DASS-21 |  |  | X |  | X |
|  | D-KEFS Condition 2 |  |  | X |  | X |
|  | WAIS-IV Digit Span |  |  | X |  | X |
|  | WAIS-IV Block Design |  |  | X |  | X |
|  | Benton Visual Retention Test |  |  | X |  | X |
|  | Boston Naming Test (15 Item) |  |  | X |  | X |
|  | Semantic Fluency (Animals) |  |  | X |  | X |
|  | Controlled Oral Word Association Test (FAS) |  |  | X |  | X |
| Biological assessment | EEG |  |  | X |  | X |
|  | Blood sample** |  |  | X | X | X |
| Safety and compliance | FBC, LFT, RFT |  | X |  | X | X |
|  | Adverse Events |  |  |  | X | X |
|  | Compliance |  |  |  | X | X |
| Note: D-KEFS = Delis-Kaplan Executive Function System; DASS-21 = 21-item Depression, Anxiety, Stress Scale; F2F = Face to Face; FAQ = Functional Activities Questionnaire; GDS = Geriatric Depression Scale; IQCODE = Informant Questionnaire on Cognitive Difficulties; MoCA = Montreal Cognitive Assessment; RAVLT = Rey Auditory Verbal Learning Test; QOL-AD = Quality of Life in Alzheimer’s Disease; TICS-M = Telephone Interview for Cognitive Status – Modified Version; TMT = Trail Making Test; ToPF = Test of Premorbid Function; WAIS-IV = Weschler Adult Intelligence Scale® Fourth Edition; WMS-IV = Weschler Memory Scale® Fourth Edition.  * Screening assessments that are also secondary outcomes  ** Blood sample collected for APOE-e4 genotyping and for markers that will be reported elsewhere as sub-studies (e.g., inflammatory markers). | | | | | | |

| **Table s4.** Online protocol implemented for the 10 trial participants. List of adaptations (if any) made to primary and secondary outcome measures to facilitate online administration for those tested during the COVID-19 public health orders. | |
| --- | --- |
| **Test name** | **Comments for Online Administration** |
| 1. Logical Memory – Immediate Recall | Verbal administration with no changes or additional processes required. |
| 1. QOL-AD | Editable PDF of survey created. Displayed to participant whilst screen sharing. |
| 1. DASS-21 | Editable PDF of survey created. Displayed to participant whilst screen sharing. |
| 1. FAQ – self report | Editable PDF of survey created. Displayed to participant whilst screen sharing. |
| 1. MoCA | PDF created which only has the Visuospatial/Executive and naming subtests. Displayed to participant whilst screen sharing. Remaining elements verbally administered with no changes or additional processes. |
| 1. Logical Memory – Delayed Recall | Easy verbal administration with no changes or additional processes required. |
| 1. RAVLT | Easy verbal administration with no changes or additional processes required. |
| 1. D-KEFS Conditions 2 and 4 | Testing stimuli scanned and displayed to participant whilst screen sharing using the interactive white board function in Zoom ®. |
| 1. Block Design | Test omitted as it can only be conducted face-to-face. |
| 1. RAVLT Delayed Recall (~ 20 min) | Verbal administration with no changes or additional processes required. |
| 1. Rey Complex Figure Test – Copy | Displayed to participant whilst screen sharing. |
| 1. 15-item Boston Naming Test | Testing stimuli scanned and displayed to participant whilst screen sharing. |
| 1. COWAT (F/A/S) | Verbal administration with no changes or additional processes required. |
| 1. Semantic Fluency – Animals | Testing stimuli scanned and displayed to participant whilst screen sharing verbal administration with no changes or additional processes required. |
| 1. Rey Complex Figure Test – Delayed Recall | Displayed to participant whilst screen sharing. |
| 1. Digit Symbol Coding | Displayed to participant whilst screen sharing. However, speed was likely compromised given different writing surface |
| 1. Digit Span | Verbal administration with no additional processes required |
| 1. Benton Visual Recognition Test | Testing stimuli scanned and displayed to participant whilst screen sharing |
| *Note:* D-KEFS = Delis-Kaplan Executive Function System; DASS-21 = 21-item Depression, Anxiety, Stress Scale; FAQ = Functional Activities Questionnaire; MoCA = Montreal Cognitive Assessment; RAVLT = Rey Auditory Verbal Learning Test; QOL-AD = Quality of Life in Alzheimer’s Disease. | |

**Section s1.** Eligibility Criteria.

Participants had to meet ALL of the following inclusion criteria prior to study entry to be eligible for enrolment:

- ≥60 years of age
- Subjective AND objective memory complaints:
  - Subjective memory complaint or other cognitive difficulties corroborated by an informant as measured by the Informant Questionnaire on Cognitive Decline in the Elderly (IQCODE) (Score ≤4)
  - Objective memory complaint measured on the Rey Auditory Verbal Learning Test (RAVLT; 1.5 SDs below the age and education-matched mean or that which would be expected based on premorbid function)
- Confirmed diagnosis of MCI core clinical criteria according to the National Institute on Aging-Alzheimer’s Association (NIA-AA) working group guidelines
- Scoring 18-26 on the Montreal Cognitive Assessment (MoCA)
- No or minimal impairment in activities of daily living: scoring <9 on the Functional Activities Questionnaire (FAQ)
- No severe depression: scoring ≤19 on the 30 item Geriatric Depression Scale (GDS)
- Agreement to sign the informed consent.

Participants that met ANY of the following exclusion criteria were excluded from the trial:

- Diagnosis of dementia
- Diagnosed psychiatric disorders including (but not limited to): dissociative disorder, obsessive-compulsive disorder, personality disorder, schizophrenia, bipolar disorder
- History of drug and alcohol dependence or substance-related disorders
- History of seizures
- Head trauma with loss of consciousness
- Left-handedness
- Allergic to at least 1 ingredient of SLT
- Current use of ginkgo, ginseng, or saffron
  - Participants taking these herbal medicines were asked to cease use for an 8 week washout period prior to the trial in order to avoid exclusion
- History of severe renal and hepatic disorders.

**Section s2.** APOE-ε4 Genotyping.

A tourniquet was applied to the participant’s arm (over the bicep muscle, proximal to the elbow), and after instruction to “pump the fist” three times, the prominent veins were located and palpated on the ante-cubital area. Warm packs were used if locating a prominent vein proved difficult. A 23 G × 19 mm butterfly needle with lure, 10 mL EDTA vacutainer collection tubes, and a vacutainer standard holder (Becton, Dickinson, and Company, USA) were used to collect two 10 mL tubes of whole blood from each participant. Blood samples were weighed and centrifuged at 23 ^o^C for 6 minutes at 4000 rpm to separate the plasma, which was aliquoted (1000 μL) into 1500 μL Eppendorf tubes. The plasma and the remaining erythrocytes and buffy coat were stored separately at -80 ^o^C until trial completion.

Genomic DNA (gDNA) was purified from 200 µL of extracted blood/buffy coats using a commercial purification kit (Promega ReliaPrep™ Blood gDNA kit Cat. # A5081) according to the manufacturer instructions and quantified using a microvolume nanodrop Spectrophotometer (Denovix Ds-11, Gene Target Solutions). Where gDNA extraction was unsuccessful, a second extraction was performed using an alternate kit (GeneJET DNA Purification Mini Kit Cat. #K0781, Thermofischer Scientific). Following raw gDNA extraction, all samples were adjusted with nuclease-free water until gDNA concentration was within the 15–60 ng/µL range and all samples had a 260:280 ratio between 1.5 and 1.8.

Taqman SNP genotyping assays detecting the rs7412 and rs429358 APOE-ε SNPs (Cat. # 4351379, Thermofischer, Australia) were used in combination with the Taqpath ProAmp Mastermix (Cat. # A30865, Thermofischer, Australia) to setup the PCR reactions according to the manufacturer’s instructions. The prepared gDNA/reactions were subsequently run on a MyGo Pro real-time PCR (InVitro Technologies) using the following cycling conditions; pre-read 60 °C 30 s cycle hold; initial denature 95 °C 5 min cycle hold; denature 95 °C 15 s cycle 40; anneal/extend 60 °C 60 s cycle 40; post-read 60 °C 30 s cycle hold. Once thermocycling was complete for both allele assays, determination was deemed through the combination of both alleles using the onboard software (v3.5.21).
